# Indents

**Published:** 1914-09-15

**Authors:** 


### INDENTS.

**☞Brief Articles of Technical or Scientific Value will receive notice and comment. Contributions invited.**

*Easy Method of Coating Models with Tin Foil Before Vulcanizing.*

After the case is packed with rubber, boiled, and the flask and the bolts have been tightened, the case is allowed to set a minute or two, and then the bolts are loosened. The halves of the flask are separated, the piece of starched linen that covers the rubber when bought being used as a separating medium, so that the upper portion will not adhere to the model. The excess of rubber is trimmed off. On the model in the other half of the flask, a whole sheet of No. 10 tin foil is burnished. No attempt should be made to trim off the excess of tin foil, for in so doing one is liable to distort the rest, and the surplus will not hurt, but rather provides a vent, allowing the excess rubber to ooze out. After the tin foil is well burnished the half of the flask containing the teeth and rubber is placed in position over the tin-foiled side. The flask is then closed and boiled, the bolts are tightened, and the case is then ready to be vulcanized.—C. S. Kile, *Western Dental Journal.*

*Early Orthodontia.*

It is a recognized fact that rhinology to-day looks to dentistry for aid in establishing normal breathing. By stimulating the growth of the bones and widening the upper arch, the floor of the nose is enlarged. The meati are enlarged, septal curvatures are modified and restored function encourages growth of the nose. We who are custodians of the mouth are also responsible for the condition of the nose. It is self-evident, therefore, that we should guard against the formation of spurs, enlarged turbinates and pathological conditions, by bringing about normal occlusion as soon as is feasible, instead of waiting until the full complement of the teeth erupt.—J. A. Cameron Hoggan, *Dental Cosmos.*

*"Clean" Teeth.* We have in times past made the statement to our patients that "clean teeth will not decay." And this is perfectly true, but the only way we can have an absolutely clean tooth is to remove it from the mouth, and keep it in a clean place. Cleanliness of the teeth in the mouth is but a relative term, and, even though a tooth be polished ever so thoroughly, it is immediately covered and bathed in an infection and acid-producing fluid which may begin at once the early stages of caries. Especially is this true in some mouths, where we see that the greatest care by the patient and the operator is not able to prevent the occurrence of caries.—Russel W. Bunting, *Dental Cosmos.*

*Responsibility In Dental Malpractice.* The horoscope drawn by Dr. Ottolengui in *Items of Interest* should have a wider range than that of the immediate readers of that journal, and I quote from *The Journal of the Allied Dental Societies*, in which it appears, "At present, the possibility that arthritis and other diseases may have their origin in septic areas about the roots of filled teeth is scarcely more than a probable hypothesis. But it is not impossible that within a few years cold science will have changed this from a theory to a proven fact. If so, then in a case of arthritis traced by a physician directly to an improperly filled root canal, there is but little doubt that the dentist who imperfectly filled the root, thus irritating the septic lesion, may be mulcted in heavy damages as a malpractitioner."

At first sight the proposition seems an alarming one, but it carries its own healing along with it. The conscientious dentist can and will only do his best, and be his efforts at root-filling what they may, by the laws of a chance, which are by no means random laws, circumstances may fight against his attaining perfection and prevail. Granted that a case of arthritis existed in the same individual with an imperfectly filled root, and granted that the physician and patient were above suspicion, granted that the dentist was "mulcted in heavy damages," there is the satisfaction in feeling fairly certain that the case would be an unique one, as no dentist would be foolish enough to lay himself open in the future to such a charge, and to such a penalty. In plain words, roots would cease to be filled, and thousands of teeth would be remorsely extracted which under present conditions would have done excellent service. and by their good work possibly saved the patient from arthritis and other afflictions.—Oswald Fergus, in *Dental Record.*

*Many Seals Sold in Ohio.* According to the report of the National Association for the Study and Prevention of Tuberculosis more than 44,000,000 Red Cross Christmas Seals were sold during the recent campaign in the United States. Of these 2,838,892 were sold in Ohio, placing this state second in rank to New York.

*Preserving Mercury.* If preserved in a glass bottle under alcohol mercury remains permanently pure and bright. If the bottle is reversed, only mercury flows out, the alcohol remaining above the heavier metal.—*Cosmos.*

*The Use of Floss Silk in Daily Dental Toilet.* There is no doubt that floss silk is the most efficient agent, if properly used, for cleansing the lower anterior teeth and preventing the accumulation of tartar at that point. For its proper and effective use I have for many years been teaching my patients the following simple method which if followed, has proved entirely satisfactory: The thread is passed entirely around the tooth, the ends are crossed, and drawn tight, and first one end and then the other is pulled, thus wiping the entire circumference of the tooth. Each tooth is treated separately, at first before a mirror until the user is familiar with the operation. When giving my patient the first lesson I use a black thread, so that the procedure may be seen more easily.—D. W. Barker, *Oral Hygiene.*

*The American Red Cross in European War.* At a joint meeting of the International and War Relief Boards of the American Red Cross held in Washington, August 5th, it was decided formally to tender the services of the society to the warring nations, and plans were initiated for the immediate issuance of appeals for relief funds and for placing medical officers and nurses trained for the work at the disposal of the forces. Announcement has been made that a fully equipped hospital ship carrying physicians, nurses and medical supplies will be sent to Europe, sailing under the Red Cross Flag, and under protection of the Geneva Convention and Hague Treaties.

*Subscription Swindler at Work.* Attention is called to the operation of a swindler who recently has been operating among doctors and dentists, soliciting subscriptions for which he is not authorized. His offers concern generally a combined subscription to *The Literary Digest* and such medical publications as a dictionary, medical clinics and other medical works. Numerous aliases are used in signing receipts. He is described as about 21 years old, 6 feet tall, weighing about 180 pounds. Has black hair, very dark and large black eyes, a deep falsetto voice, and somewhat pale skin. A reward of $25 has been offered for his arrest. Communications should be sent to the Periodical Publishers' Association, 156 Fifth Avenue, New York City.—*Journal, A. M. A.*, August 15, 1914.

*Antiseptic Varnish for Surgeons; Mastisol, Steresol.* In our February number (p. 155), mention was made of a bactericidal varnish—*steresol*—composed of Peru balsam, aceptic acid, castor oil, and alcohol; this to serve surgeons in lieu of rubber gloves when operating or in gynecologic work.

We now happen across another formula of a similar nature and claimed to yield a preparation giving fully as good satisfaction as a certain German proprietary marketed under the name of *mastisol.* This combination was developed by Dr. Borchardt (*Bruns. Beitr. z. Chir.*, Bd. 87, No. 2), who had been using the mastisol (but found it rather costly), and is as follows: Finest mastic, Gm. 40; purest benzol, Gm. 60; castor-oil, gtt. 20. Dissolve, let stand, then filter. (Do not confuse benzol with benzine.)

This varnish markedly inhibits the development of strepto-and staphylococci. If the brush with which it is laid on becomes infected, it can be sterilized by immersing it in the liquid for an hour. This varnish, the author suggests, should be carried in the surgical bag. It may be strengthened by adding 3 to 6 percent of iodine. In case of ablations, the varnish may be applied after painting the field with iodine.—*Clinical Medicine.*

*The Fatalities from Salvarsan.* The eight deaths following the untradural administration of neosalvarsan-serum for nerve syphilis in one hospital in Los Angeles, all occurring within forty-eight hours, have brought home to the medical profession more strikingly than ever before the fact that salvarsan is not alone a useful drug but a dangerous one as well. At least 275 deaths are known to have followed its administration, while in all probability the total number is several times this, since nearly every physician can tell of cases which have not been reported.

In a recent book upon the subject, Wechselmann brings out the important point that very many of these deaths from salvarsan may be attributed to the combined or alternated use of salvarsan and mercury. He declares that anything delaying the excretion of an arsenical preparation brings out the toxic action of that drug and may cause a fatal issue. For this reason, he strongly advises against administering salvarsan *following* the giving of mercury, and especially of metallic mercury, inasmuch as the latter drug interferes with normal kidney function and thereby delays excretion.

The moral of this observation is, that before using salvarsan a careful chemical and microscopical examination of the urine should be made in every instance. Also, salvarsan and mercury in large dosage should never be employed together. And, finally, when the combined treatment seems to be indicated, the arsenical preparation always should be given first, and never, under any circumstances, after a course of treatment with a mercurial.

There is no denying the value of salvarsan, and we have no disposition to minimize the importance of Ehrlich's great discovery. Yet time has only served to teach the thoughtful physician caution in the use of this remedy, the injudicious administration of which we now know to be fraught with dangers little dreamed of by the average physician. Mercury still stands first as a specific remedy for syphilis. Not only is it safe; but its curative value is undoubtedly greater than that of salvarsan. The latter drug has its own place—but, for the present, it may well be left to men who are thoroughly familiar with its field and know best how to use it.

He who goes safely will go far. Our advice to the general practitioner is keep in mind that popular maxim, "safety first," even in treating syphilis.—*Clinical Medicine.*

*Anesthesia of the Mandible.* The author recommends local anesthesia for operations in the mandible and obtains a thorough insensibility of the region by means of injections of novocain into the trunk of the inferior dental nerve at its entrance into the inferior dental canal. His method consists in reaching the nerve from a point about one centimeter in front of the mandibular angle. The needle which must be, at least, $4\frac{1}{2}$ centimeters in length is introduced at this spot and gradually forced upwards in contact with the internal surface of the ascending ramus, and parallel to its posterior border. At a distance of about $4\frac{1}{2}$ centimeters, the nerve is reached as shown by the momentary shooting pain in the teeth felt by the patient. The syringe is then held rigidly with one hand while with the other the contents are forced at once into the tissues. The anesthetic effect is sometimes instantaneous, occasionally it will appear after ten minutes but generally insensibility is complete in from two to five minutes. The anesthesia lasts from one and one half to two hours and enables the operator to extract teeth painlessly, and to curette, resect or suture the mandible. The method of reaching the nerve from a point in the mouth posterior to the third molar or externally the needle traveling at right angles to the trunk of the nerve is severely condemned by the author.—Robert Danis, in *Pacific Gazette.*

*Supporting Loose Teeth.* In cases where the peridental membrane is weakened, where with extraordinary or ordinary stress these teeth can be moved, then if they are connected by inlays soldered together there is going to be a good deal more service rendered that patient from a masticatory standpoint than if those teeth were restored and the inlays not attached. I know this because I am doing it in my own practice, and rendering good service. A case has been in the mouth for three or four years and the patient is getting good service from the teeth in that manner and I am satisfied that the teeth would have been lost long ago had they not been connected in some such manner. The point that is immediately brought out in objection to the connection of the inlays by soldering is this, that the free movement of the roots of the teeth is restricted and more or less irritation will follow, and possibly result in the loss of the teeth. Now it is those cases where the peridental membrane is weak where this condition is necessary, and we find when a case has been treated in this manner and the inlays have been in place for two, three or four years, they are much firmer than when the inlays were first set in place. I speak thus strongly because of the very satisfactory results I have had.—Dr. Prothro, in *Dominion Journal.*

